# Compliance With NICE-Recommended Physical Health Monitoring for Children and Young People Prescribed Antipsychotic Medication Within Nottinghamshire CAHMS Head-to-Head Community Service: A Retrospective Audit

**DOI:** 10.1192/bjo.2026.11848

**Published:** 2026-06-30

**Authors:** Florence Stanger, Sree Nair

**Affiliations:** 1 Nottingham University, Nottingham, United Kingdom; 2 CAHMS, Nottinghamshire, HC NHS Trust, Nottingham, United Kingdom

## Abstract

**Aims:**

Antipsychotic medications are widely prescribed in child and adolescent mental health services (CAHMS) and carry recognised metabolic and cardiovascular risks. National Institute for Health and Care Excellence (NICE) guidance outlines minimum standards for physical health monitoring before and during antipsychotic treatment. This audit aimed to assess compliance with NICE-recommended physical health monitoring for children and young people prescribed antipsychotic medication within a CAHMS Head-to-Head (H2H) community service.

**Methods:**

A retrospective audit of electronic patient records (Rio) was conducted. All patients under the CAHMS H2H team prescribed antipsychotic medication were identified. Physical health monitoring was assessed at baseline, six weeks, twelve weeks, and at six-monthly intervals thereafter. Six-monthly monitoring was analysed per eligible monitoring window, recognising that patients commenced treatment at different times. Patients admitted to inpatient wards when monitoring was due were excluded. Patients who had not yet reached eligibility for six-monthly monitoring were excluded from six-monthly denominators. Monitoring performed during medication titration or switching was outside the scope of this audit. Descriptive statistics were used to calculate completion rates.

**Results:**

Eleven patients were identified; two were excluded, leaving a final audit cohort of nine patients aged 15–18 years. Complete baseline physical health monitoring was documented in 0% of patients, although 44.4% had at least one baseline parameter recorded. Six-week weight monitoring was completed in 22.2% of patients, and complete twelve-week monitoring (weight, blood pressure/heart rate, and blood tests) in 22.2%. Seven patients were eligible for six-monthly monitoring, contributing a total of 25 eligible six-monthly monitoring windows. Completion rates for individual NICE-recommended parameters across these windows were low (height 20.0%, weight 20.0%, blood pressure/heart rate 16.0%, blood tests 20.0%, waist circumference 0%), with no window containing all recommended measures. Despite this, all patients had evidence of some ongoing physical health monitoring while prescribed antipsychotic medication, although this did not consistently occur within NICE-specified timeframes. No adverse physical health outcomes related to delayed or out-of-window monitoring were identified.

**Conclusion:**

Formal compliance with NICE-recommended monitoring schedules was low when assessed against strict guideline timeframes. However, all patients received ongoing physical health monitoring and remained clinically safe. These findings highlight the challenges of implementing rigid monitoring schedules within CAHMS and support the need for systems that promote consistent, well-documented monitoring while accommodating real-world clinical and family circumstances.

